# An online dynamic nomogram for predicting acute kidney injury after endovascular therapy in acute ischemic stroke

**DOI:** 10.1186/s12882-026-04773-9

**Published:** 2026-01-27

**Authors:** Kaiwei Cai, Hongyu Qiao, Ka Lung Chan, Qihuan Liu, Li’an Huang, Yusheng Zhang, Min Guan, Bing Yang, Anding Xu, Jun Lyu, Dan Lu

**Affiliations:** 1 Department of Neurology & Stroke Center, Key Lab of Basic and Translational Research of Pan-vascular Diseases, The First Affiliated Hospital of Jinan University, Guangzhou, 510632 China; 2 Clinical Neuroscience Institute, The First Affiliated Hospital of Jinan University, Guangzhou, 510630 China; 3 Department of Neurology, Neuromedicine Center, The University of Hong Kong-Shenzhen Hospital, Shenzhen, China; 4 Department of Clinical Research, The First Affiliated Hospital of Jinan University, Guangzhou, 510630 China; 5 Guangdong Provincial Key Laboratory of Traditional Chinese Medicine Informatization, Guangzhou, 510630 China

**Keywords:** Acute kidney injury, Acute ischemic stroke, Endovascular therapy, Nomogram, Prognosis

## Abstract

**Objective:**

There is still a lack of effective means for comprehensively and accurately evaluating the risk of acute kidney injury (AKI) after endovascular therapy (EVT) in patients with acute ischemic stroke (AIS). Therefore, this study aimed to develop and validate a nomogram for accurately predicting the risk of AKI after EVT in patients with AIS.

**Methods:**

673 patients were included in our study, comprising 324 in the training cohort, 140 in the internal validation cohort, and 209 in the external validation cohort. The nomogram was developed based on the variables screened using the least absolute shrinkage and selection operator regression and multivariate logistic regression. The predictive efficiency of the nomogram was validated using both internal and external validation cohorts. Furthermore, the association between AKI and 90-day functional outcomes and mortality was analyzed based on propensity-score matching.

**Results:**

Five risk factors for AKI were identified and used to construct a nomogram: baseline National Institutes of Health Stroke Scale score, preoperative creatinine level, pneumonia, acute heart failure, and hypotension. The nomogram exhibited good predictive performance as assessed by the area under the curve, calibration plots, and decision-curve analysis. In the propensity-score matched cohorts, AKI remained significantly associated with 90-day worse neurological outcomes.

**Conclusions:**

We developed and validated an online dynamic nomogram with high predictive accuracy for predicting the risk of AKI in patients with AIS undergoing EVT, which is beneficial for clinical decision-making. Furthermore, our analysis showed that AKI was associated with poorer functional outcomes after EVT.

**Supplementary Information:**

The online version contains supplementary material available at 10.1186/s12882-026-04773-9.

## Introduction

Acute ischemic stroke (AIS) has significant mortality and disability rates, imposing substantial social and economic burdens [1, 2]. It has been demonstrated that endovascular therapy (EVT) significantly improves the rate of vascular reperfusion, thereby improving the neurological prognosis of AIS patients with large-vessel occlusion [3]. However, acute kidney injury (AKI) may occur in patients with AIS undergoing EVT due to the activation of the sympathetic nervous system (SNS) and the renin-angiotensin-aldosterone system (RAAS), the release of inflammatory factors, and the use of contrast agents [4], which is associated with reduced quality of life and a poor prognosis.

The incidence of AKI reportedly ranges from 7% to 20% in patients with AIS undergoing EVT [5–7]. AKI is characterized by a sudden deterioration in renal function, manifested by an acute increase in serum creatinine or a decrease in urine output, which can lead to water and sodium accumulation, electrolyte and acid-base disorders, and toxin accumulation, thereby increasing risks of death, end-stage renal disease, and new chronic kidney disease [8]. A meta-analysis found that AKI after stroke was significantly associated with more extended hospital stays and increased short- and long-term mortality rates [9]. In addition, AKI causes structural damage to the kidney. Even if creatinine subsequently returns to normal levels, the changes in renal structure may persist, leading to long-term risks of renal and nonrenal complications [10]. Therefore, rapid identification of high-risk AKI patients and timely pre-intervention to prevent the occurrence and development of AKI are crucial for improving the prognosis of patients. However, there is currently a lack of risk assessment tools to quantify the risk of AKI, which hinders the implementation of targeted early intervention measures.

Nomograms are now essential tools for accurately predicting the probability of clinical events and are widely used in various fields [11]. In this study, we aimed to develop a user-friendly and effective nomogram for accurately assessing the risk of AKI in patients undergoing EVT, thereby providing scientific guidance for early individualized prevention strategies. In addition, given that the association between AKI and functional outcomes of stroke remains controversial [5, 12], we also investigated the relationship between AKI and functional outcomes in patients undergoing EVT using the propensity-score matching (PSM).

## Data sources and methods

### Patient selection

This retrospective study enrolled patients with AIS undergoing emergency EVT at the Stroke Center of the First Affiliated Hospital of Jinan University in China from January 2017 to April 2024. The inclusion criteria were: (1) age ≥ 18 years, (2) AIS diagnosis, and (3) EVT initiation within 24 h of symptom onset. The diagnosis of large vessel occlusion (LVO) was determined based on clinical symptoms and the results of computed tomography angiography (CTA) or magnetic resonance angiography (MRA). Key exclusion criteria comprised other encephalopathies, end-stage renal disease, chronic dialysis, severe liver disease, missing preoperative creatinine data, previous stroke with residual disability (mRS ≥ 2), and discharge or death within 24 h post-EVT.

Patients enrolled between January 2017 and November 2022 were randomly assigned to the training and internal validation cohorts at a ratio of 7:3 [13]. Patients were enrolled as the external validation cohort between December 2022 and April 2024 [14]. The training cohort was used to build predictive models, while the internal and external validation cohorts were used to test the predictive performance of the models.

The study was carried out in accordance with the Declaration of Helsinki. The Institutional Ethics Committee of the First Affiliated Hospital of Jinan University reviewed and approved the study protocol (Approval number: KY-2024-194).

### Data collection

Baseline demographic characteristics, preoperative clinical data (including comorbidities and preoperative laboratory data), surgical data, medical treatment data, and main complications data were collected in detail from the electronic medical records of the patients. The collected baseline demographic characteristics and preoperative clinical data included age, gender, preoperative systolic blood pressure (SBP), diastolic blood pressure (DBP), vascular occlusion site, baseline National Institutes of Health Stroke Scale (NIHSS) score, and the presence of thrombolysis, hypertension, diabetes, atrial fibrillation (AF), coronary heart disease (CHD), cancer, and baseline renal insufficiency (defined as an estimated glomerular filtration rate (eGFR) of < 60 ml/min/1.73 m^2^ at admission) [15]. eGFR was calculated according to the CKD-EPI equation [16]. Preoperative laboratory data included platelet, white blood cell, neutrophil, red blood cell, and lymphocyte counts, and hemoglobin, creatinine, and blood urea nitrogen levels. Surgical data comprised the time from onset to puncture, time from puncture to recanalization, dose and type of contrast agent, and successful reperfusion defined as an mTICI (modified thrombolysis in cerebral infarction) grade of 2b or higher. Medical treatment data, such as antiplatelet agents, anticoagulants, statins, edaravone, antibiotics, mannitol, diuretics, angiotensin-converting enzyme inhibitors, and angiotensin II receptor blockers, were collected. The collected main complications data included subarachnoid hemorrhage (SAH), symptomatic intracranial hemorrhage (sICH; defined according to ECASS‑III criteria [17]), pneumonia, acute heart failure (AHF), and hypotension (defined as SBP < 90 mmHg for more than 30 min) [18].

The primary outcome was the occurrence of AKI within 7 days after surgery. According to KDIGO guidelines [19], AKI is diagnosed based on an absolute increase in serum creatinine of ≥ 26.4 µmol/L within 48 h or an increase of ≥ 50% from baseline within 7 days. It is important to note that all predictor variables were recorded at time points strictly preceding the diagnosis of AKI. The secondary outcomes were 90-day mortality and adverse functional outcomes. An adverse functional outcome was defined as a modified Rankin Scale (mRS) score of 3 or more.

### Statistical method

The required sample size was calculated using the pmsampsize package in R statistical software and the formula developed by Riley et al. [20]. Based on the findings of our pilot study, the incidence of the outcome events was 13%, the expected number of predictors was six, and the C statistic was 0.85. The minimum sample size required for the training cohort was 254 patients, with 5.5 events per predictor.

Categorical variables were described as number and percentage values, and continuous variables were expressed as mean ± standard deviation values or median and interquartile range values. Differences between groups were tested using the chi-square test or Fisher’s exact test for categorical variables and the independent-sample t-test or Wilcoxon rank-sum test for continuous variables. Missing data were supplemented by multiple imputation using the R package “mice”, with variables being deleted when they had > 20% missing values.

Potentially significant risk factors were preliminarily screened in the training cohort using the least absolute shrinkage and selection operator (LASSO) implemented with the R package “glmnet”. LASSO regression is a compressed estimation method that selects variables based on a parameter λ. In the process of regression, the regression coefficient of a stronger factor is compressed to 0 later with the increase of the λ value. The factors with a high correlation with the occurrence of AKI were chosen after 10-fold cross-validation, with the λ value within one standard error of the minimum criteria. Variables selected by LASSO were subsequently entered into a multivariable logistic regression model. Predictors with a *P*-value < 0.05 were retained as independent predictors in the final model. Before fitting the final multivariate logistic regression model, the linearity of the logit for continuous variables was assessed using restricted cubic splines (RCS) with 3 knots (placed at the 10th, 50th, and 90th percentiles). This knot selection aimed to strike a balance between flexibility in detecting potential nonlinear patterns and avoiding the risk of overfitting. Nonlinearity was tested using the Wald test; a *P*-value > 0.05 for nonlinearity supported their inclusion as linear terms. The absence of substantial multicollinearity in the final model was confirmed by calculating Variance Inflation Factors (VIF), with all values being below 5. Finally, a nomogram was constructed based on the final set of independent predictors to estimate the risk of AKI following EVT in patients with AIS.

The predictive performance of the nomogram was evaluated by analyzing its discrimination ability, calibration, and clinical practicability, and was further verified in the validation cohort. The discrimination ability of the nomogram was assessed by the area under the curve (AUC) of the receiver operating characteristic curve (ROC), with a value of ≥ 0.7 considered to indicate a good ability. The calibration of the nomogram was evaluated using calibration plots and Hosmer-Lemeshow tests (HL tests). A high coincidence of the ideal reference line and the actual prediction performance curve in the calibration plot indicates good prediction efficacy, and a P-value > 0.05 in the HL test suggests no statistically significant evidence of poor fit. The clinical applicability of the nomogram was assessed by quantifying the net benefit under different threshold probabilities using decision-curve analysis (DCA). To further assess model robustness and potential overfitting, internal validation was conducted via bootstrap resampling with 1000 repetitions in the training cohort, yielding a corrected AUC and a bootstrap-calibrated curve.

In addition, the 90-day mortality and adverse outcomes of patients with AKI were assessed by dividing the total population into AKI and non-AKI groups. The confusion bias between the two groups was minimized by applying PSM using the R package “MatchIt”, with 1:1 nearest-neighbor matching and a caliper width of 0.2. The following baseline covariates, selected for their clinical relevance to the outcomes of interest, were included in the propensity model: age, sex, baseline NIHSS score, vascular occlusion site, thrombolysis, hypertension, diabetes, CHD, AF, cancer, baseline renal insufficiency, the time from onset to puncture, the time from puncture to recanalization, successful reperfusion, SAH, sICH, pneumonia, AHF, and hypotension. Covariate balance before and after matching was assessed using standardized mean differences (SMD), with SMD < 0.10 indicating adequate balance. The improvement in balance was visually summarized using a Love plot.

To address residual confounding from variables that remained imbalanced after matching (SMD > 0.10), multivariable regression analysis was applied to the matched cohort. The association between AKI and the full ordinal distribution of the 90-day modified Rankin Scale (mRS, scores 0–6) was examined using multivariable ordinal logistic regression. The proportional odds assumption of this model was tested and verified. For binary outcomes (90-day mortality and adverse functional outcome), multivariable logistic regression was used to assess their relationship with AKI.

All statistical tests were two-tailed, and a *P*-value < 0.05 was considered statistically significant. Statistical analyses were performed using R statistical software (version 4.2.2).

## Results

### Baseline characteristics

**Fig. 1:**
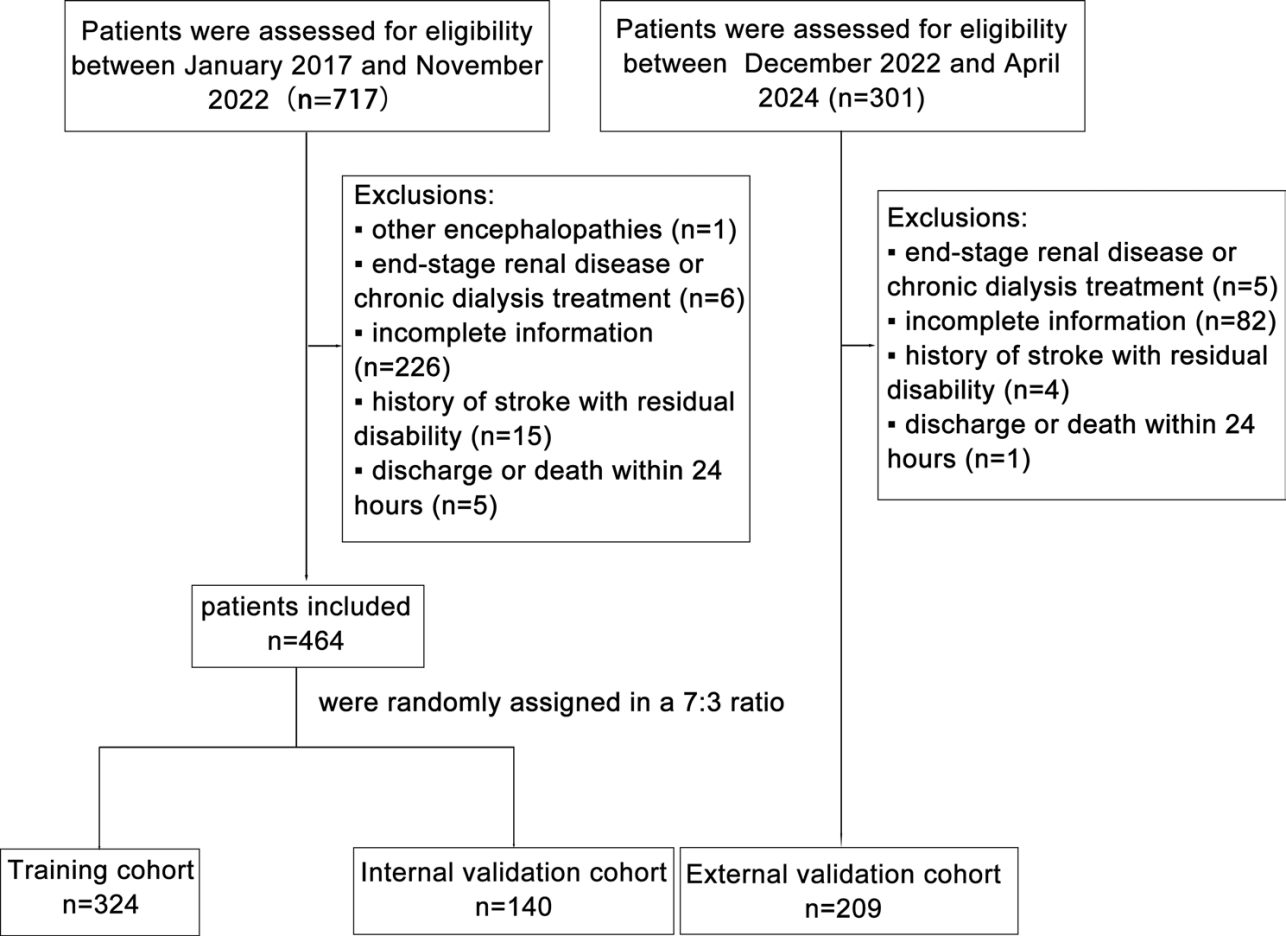
Flowchart of patient inclusion and exclusion

The patient inclusion and exclusion flowchart was presented in Fig. 1. A total of 673 patients were enrolled in this study based on the inclusion and exclusion criteria. 464 patients were enrolled in this study between 2017 and 2022, with 324 randomly assigned to the training cohort and 140 to the internal validation cohort. 209 were enrolled in this study from 2022 to 2024 and assigned to the external validation cohort. In the training cohort, the median age was 65 (interquartile range [IQR] 55–76) years, and the median baseline NIHSS score was 16 (IQR 11–21). The majority of patients were male (67.0%). The most common comorbidities were hypertension (67.3%), followed by AF (36.1%), diabetes (30.6%), and baseline renal insufficiency (21.0%). The most common complication was pneumonia (38.9%), followed by AHF (12.3%), and hypotension (9.9%). The rates of preoperative CTA implementation across cohorts are detailed in Supplementary Table S1, with the external validation cohort showing a significantly higher CTA rate. The incidence of AKI was 14.8%, 12.1%, and 9.1% in the training cohort, internal validation cohort, and external validation cohort, respectively. Specific AKI staging and time of onset are provided in Supplementary Table S2. The baseline characteristics of the training cohort and internal validation cohort were comparable. The external validation cohort exhibited notable differences in multiple baseline characteristics compared to the training cohort, underscoring its suitability for evaluating the external validity of the predictive model. Detailed baseline characteristics data are shown in Table 1. The specific missing variables can be found in Supplementary Table S3.

**Table 1:** Baseline characteristics of patients with AIS undergoing EVT

Variables	Training cohort (*n* = 324)	Internal validation cohort (*n* = 140)	External validation cohort (*n* = 209)	P1	P2
Age, years	65 (55–76)	65 (56–77)	66 (56–77)	0.455	0.090
Sex, n (%)				0.542	0.826
Female	104 (32.1)	49 (35.0)	69 (33.0)		
Male	220 (67.9)	91 (65.0)	140 (67.0)		
Smoking status, n (%)	132 (40.7)	48 (34.3)	96 (45.9)	0.190	0.237
Baseline NIHSS score	16 (11–21)	15 (10.3–20)	12 (6.5–20)	0.455	0.001
Preoperative SBP, mmHg	146 (128–163)	144 (130–162)	151 (134–165)	0.600	0.505
Preoperative DBP, mmHg	86 (77–98)	82 (75–92)	86 (77-93.5)	0.075	0.871
Vascular occlusion site, n (%)				0.824	0.002
Anterior circulation	287 (88.6)	123 (87.9)	165 (78.9)		
Posterior circulation	37 (11.4)	17 (12.1)	44 (21.1)		
Thrombolysis, n (%)	72 (22.3)	33 (23.6)	32 (15.3)	0.750	0.049
**Comorbidities**					
Hypertension, n (%)	218 (67.3)	97 (69.3)	167 (79.9)	0.672	0.001
Diabetes, n (%)	99 (30.6)	52 (37.1)	66 (31.6)	0.165	0.803
CHD, n (%)	50 (15.4)	21 (15.0)	34 (16.3)	0.906	0.796
AF, n (%)	117 (36.1)	46 (32.9)	48 (23.0)	0.500	0.001
Cancer, n (%)	10 (3.1)	7 (5.0)	5 (2.4)	0.314	0.636
Baseline renal insufficiency, n (%)	68 (21.0)	28 (20.0)	47 (22.5)	0.810	0.681
**Preoperative laboratory data**					
WBC, ×10^9^/L	9.0 (7.2–11.3)	8.7 (7.3–11.9)	8.5 (7.0-10.6)	0.804	0.150
NEC, ×10^9^/L	6.7 (4.7–8.9)	6.6 (5.0-9.6)	6.0 (4.5–7.9)	0.568	0.054
LY, ×10^9^/L	1.5 (1.1–2.1)	1.5 (1.1-2.0)	1.5 (1.0-2.1)	0.962	0.598
RBC, ×10^9^/L	4.6 ± 0.7	4.7 ± 0.7	4.7 ± 0.7	0.564	0.076
HGB, g/L	138.1 (127.1–149.0)	138.0 (126.0- 148.5)	138.5 (126.2–148.0)	0.882	0.764
PLT, ×10^9^/L	208.9 (178.3- 249.7)	214.4 (174.5- 255.1)	235.0 (192.3- 276.3)	0.558	0.000
Creatinine, µmol/L	80 (67–97)	79 (68–91)	79.2 (68-94.2)	0.577	0.714
BUN, mmol/L	5.6 (4.5–7.1)	5.9 (4.7–7.2)	5.7 (4.9–7.2)	0.396	0.397
eGFR, ml/min/1.73 m^2^				0.885	0.326
≥ 90	128 (39.5)	54 (38.6)	72 (34.4)		
60–89	129 (39.8)	59 (42.1)	90 (43.1)		
30–59	59 (18.2)	25 (17.9)	45 (21.5)		
15–29	8 (2.5)	2 (1.4)	2 (1.0)		
Blood glucose, mmol/L	7.0 (6.1–8.6)	7.2 (6.0-8.9)	7.3 (6.2–9.3)	0.518	0.546
**Surgical data**					
Onset to puncture time, hours	5.9 (3.7–8.9)	6.1 (4.4–10.0)	6.2 (3.7–11.9)	0.250	0.800
Puncture to recanalization time, hours	0.5 (0.3–0.9)	0.5 (0.3–0.9)	0.5 (0.4–1.1)	0.463	0.182
Successful reperfusion, n (%)	313 (96.6)	136 (97.1)	203 (97.1)	0.988	0.737
Contrast dose, ml	150 (120–220)	160 (120–220)	160 (120–220)	0.984	0.243
Type of contrast agent, n (%)				0.298	0.000
Iopamidol	142 (43.8)	60 (42.9)	0 (0.0)		
Ioversol	124 (38.3)	62 (44.3)	57 (27.3)		
Iodixanol	58 (17.9)	18 (12.9)	9 (4.3)		
Iopromide	0 (0.0)	0 (0.0)	143 (68.4)		
**Treatment**					
Antiplatelet, n (%)	253 (78.1)	103 (73.6)	178 (85.2)	0.291	0.042
Anticoagulants, n (%)	122 (37.7)	48 (34.3)	47 (22.5)	0.489	0.001
Statins, n (%)	315 (97.2)	137 (97.9)	207 (99.0)	0.939	0.149
Edaravone, n (%)	191 (59.0)	95 (67.9)	180 (86.1)	0.070	0.001
Antibiotics, n (%)	186 (57.4)	81 (57.9)	97 (46.4)	0.928	0.013
Mannitol, n (%)	108 (33.3)	42 (30.0)	36 (17.2)	0.481	0.001
Diuretics, n (%)	100 (30.9)	33 (23.6)	37 (17.7)	0.111	0.001
ACEI/ARB, n (%)	116 (35.8)	47 (33.6)	107 (51.2)	0.644	0.001
**Complications**					
SAH, n (%)	12 (3.7)	1 (0.7)	4 (1.9)	0.138	0.237
sICH, n (%)	14 (4.3)	2 (1.4)	3 (1.4)	0.197	0.064
Pneumonia, n (%)	126 (38.9)	54 (38.6)	72 (34.4)	0.949	0.300
AHF, n (%)	40 (12.3)	14 (10.0)	13 (6.2)	0.470	0.021
Hypotension, n (%)	32 (9.9)	15 (10.7)	18 (8.6)	0.784	0.625
**Outcome**					
AKI, n (%)	48 (14.8)	17 (12.1)	19 (9.1)	0.447	0.052

Notes: *P*1 represents the *P*-value of univariate analysis between the training cohort and the internal validation cohort. *P*2 represents the *P*-value of univariate analysis between the training cohort and the external validation cohort

Abbreviations: AIS, acute ischemic stroke; EVT, endovascular therapy; AKI, acute kidney injury; NIHSS, National Institute of Health Stroke Scale; SBP, systolic blood pressure; DBP, diastolic blood pressure; CHD, coronary heart disease; AF, atrial fibrillation; WBC, white blood cell count; NEC, neutrophil count; LY, lymphocyte count; RBC, red blood cell count; HGB, hemoglobin; PLT, platelet count; BUN, blood urea nitrogen; eGFR, estimated glomerular filtration rate; ACEI, angiotensin converting enzyme inhibitors; ARB, angiotensin II receptor blockers; SAH, subarachnoid hemorrhage; sICH, symptomatic intracranial hemorrhage; AHF, acute heart failure

### Variables selection

A total of 42 variables shown in Table 1 were included in the LASSO regression analysis. Six variables, including baseline NIHSS score, preoperative creatinine level, use of diuretics, pneumonia, AHF, and hypotension, were preliminarily identified as potential risk factors strongly correlated with AKI. These six risk factors were included in the multivariate logistic regression, with the findings showing that five of them were independent significant risk factors for AKI: baseline NIHSS score, preoperative creatinine level, pneumonia, AHF, and hypotension (Supplementary Table S4).

### Nomogram construction

**Fig. 2:**
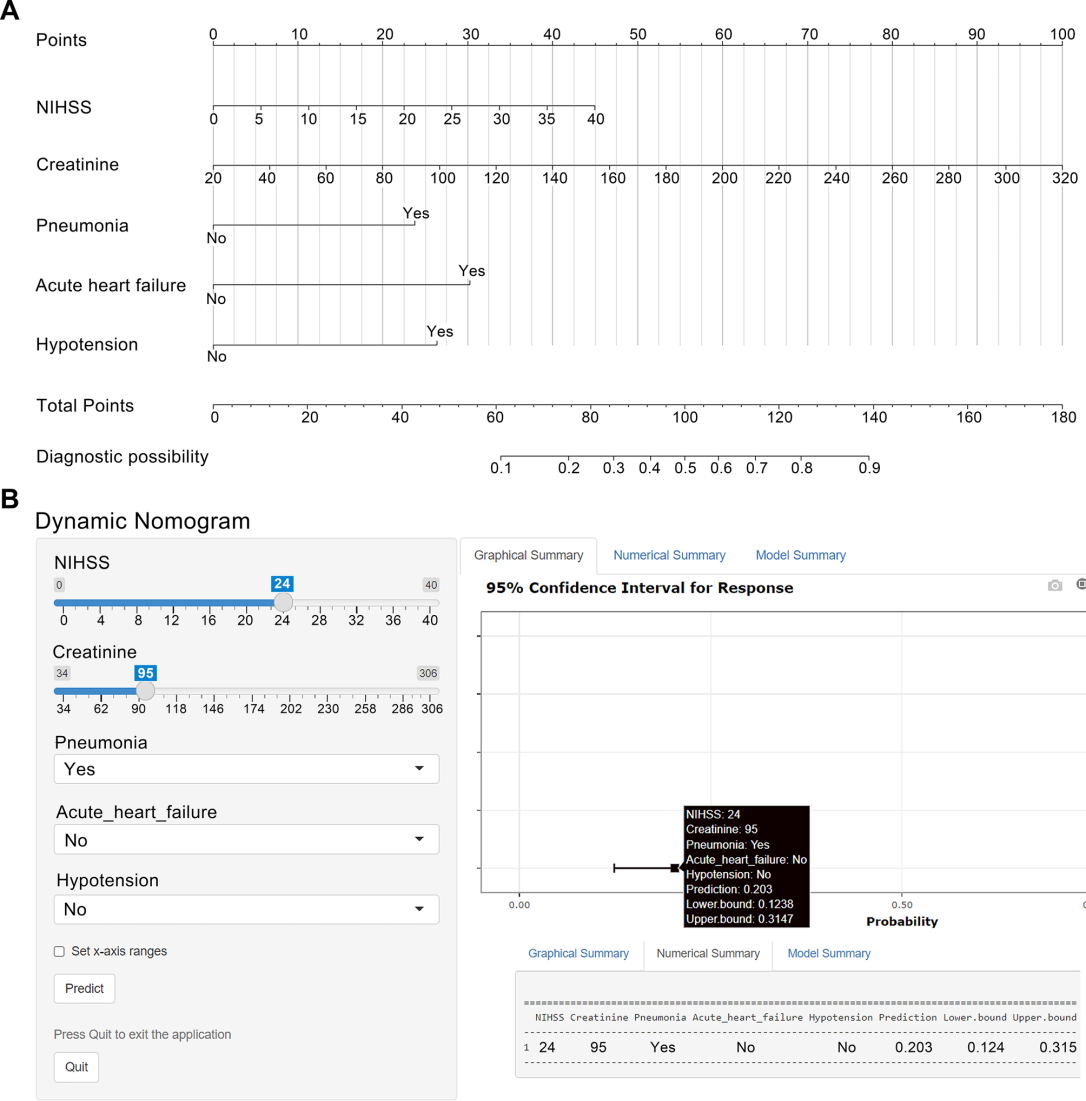
Construction of a nomogram for predicting the risk of AKI after EVT in patients with AIS. **A** The nomogram to calculate the probability of AKI in patients with AIS undergoing EVT. In the figure, the value of each predictor corresponds upward to the score of the “Points” axis. The total points generated by adding the scores of each predictor correspond downward to the probability of AKI. **B** Operation interface of the online dynamic nomogram. By simply entering five variables, the probability of AKI was automatically calculated. For example, if a patient with AIS had a preoperative NIHSS score of 24, a preoperative creatinine of 95µmol/L, pneumonia, and no AHF and hypotension, the incidence of AKI after EVT could be automatically calculated as 20.3%

These five independent risk factors were incorporated into the final multivariable logistic regression model (Table 2). Model testing showed that continuous variables met the linearity assumption (nonlinear test based on RCS, *P* > 0.05), and there was no evidence of multicollinearity among the variables (all VIF < 2; see Supplementary Fig. S1 and Supplementary Table S5). A nomogram was developed based on this model (Fig. 2A). Considering that it is time-consuming for clinicians to calculate the probability of AKI for each patient using a static nomogram, we developed a user-friendly online dynamic nomogram for clinicians to rapidly evaluate the risk of AKI by inputting the values of the predictors (Fig. 2B). The online dynamic nomogram can be accessed at https://wdynamic.shinyapps.io/DynNomapp/.

**Table 2:** Multivariate logistic regression of independent risk factors for AKI

Variables	*P*-value	OR (95% CI)
Baseline NIHSS score	0.019	1.065 (1.011–1.125)
Preoperative creatinine	0.001	1.019 (1.009–1.031)
Pneumonia	0.002	3.825 (1.636–9.571)
AHF	< 0.001	5.505 (2.353–13.008)
Hypotension	0.003	4.419 (1.601–12.044)

Abbreviations: AKI, acute kidney injury; OR, odds ratio; CI, confidence interval; NIHSS, National Institute of Health Stroke Scale; AHF, acute heart failure

### Validation of the nomogram

**Fig. 3:**
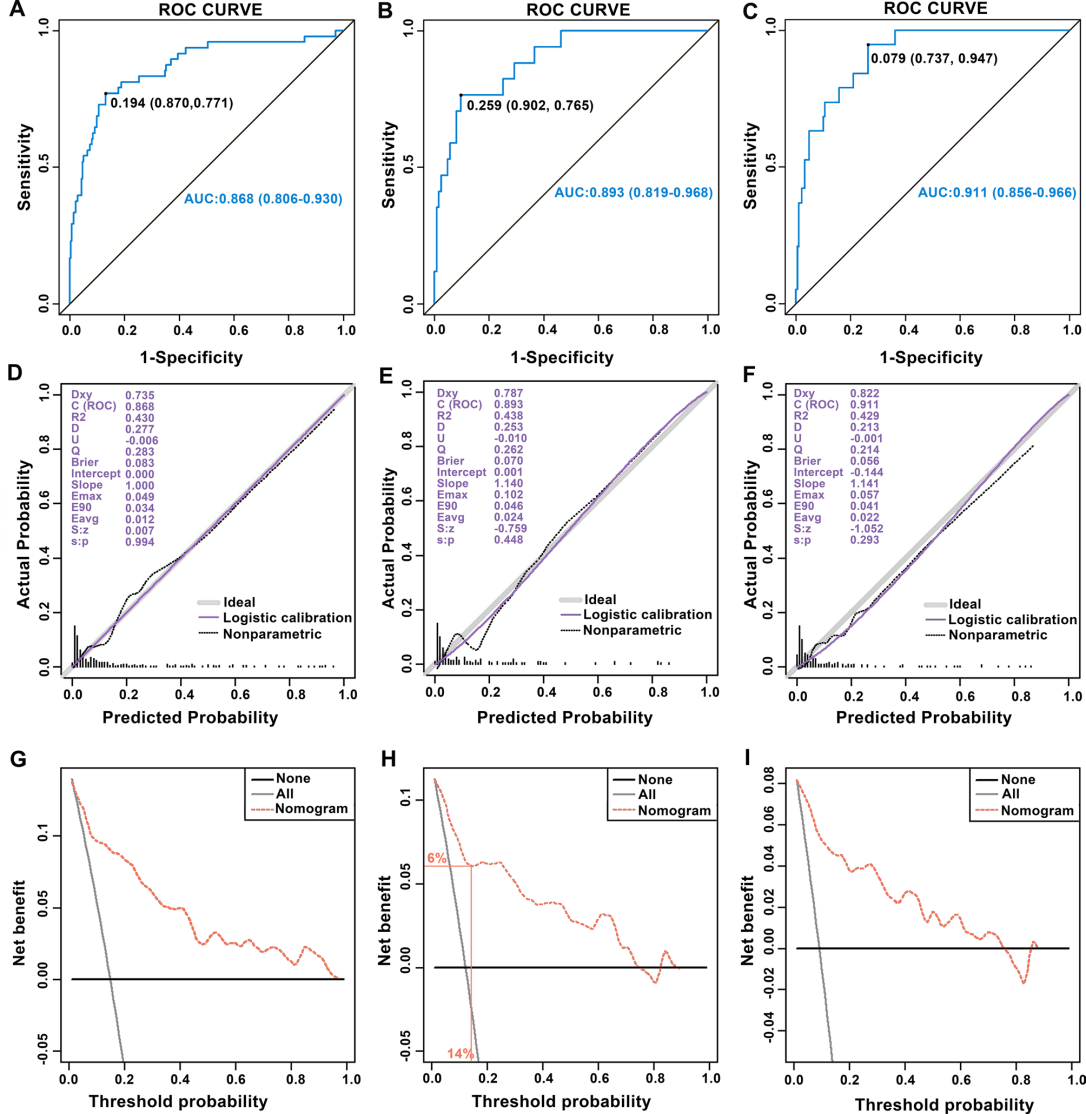
Validation of prediction performance of the nomogram. (**A**-**C**) ROC curve of the nomogram performed in the training cohort, internal validation cohort, and external validation cohort. (**D**-**F**) Calibration curves of the nomogram in the training cohort, internal validation cohort, and external validation cohort. The grey line at 45° diagonally represented the perfect prediction of the ideal model, while the purple line represented the actual prediction performance of the nomogram. The closer the purple line is to the diagonal gray line, the higher the prediction accuracy of the nomogram. (**G**-**I**) Decision curves analysis of the nomogram in the training cohort, internal validation cohort, and external validation cohort. The horizontal line represented the net benefit when all patients did not receive the intervention. The diagonal line represented the net benefit when all patients received the intervention. The yellow dashed line showed the net benefit when the intervention was decided based on the nomogram

The AUC values were 0.868 [95% confidence interval (CI) = 0.806–0.930] in the training cohort, 0.893 (95% CI = 0.819–0.968) in the internal validation cohort, and 0.911 (95% CI = 0.856–0.966) in the external validation cohort (Fig. 3A-C). These AUC results demonstrated that the nomogram had good discrimination ability. Furthermore, calibration plots showed that the actual prediction performance curves of the nomogram essentially coincided with the ideal reference line in these cohorts (Fig. 3D-F). The *P*-values in the HL tests for the training, internal validation, and external validation cohorts were 0.241, 0.829, and 0.962, respectively, indicating no statistically significant evidence of miscalibration. To rigorously assess potential overfitting, we performed bootstrap internal validation with 1000 repetitions in the training cohort (Supplementary Fig. S2). This analysis yielded a bootstrap-corrected AUC of 0.857 (95% CI: 0.796–0.923), indicating minimal optimism (optimism = 0.011). The bootstrap-corrected calibration also demonstrated excellent agreement with the ideal line (HL test = 0.214). These results indicated that the probability of AKI predicted by the nomogram was in excellent agreement with the actual probability of AKI being observed.

Finally, we evaluated the clinical utility of the nomogram using DCA. In each cohort, the model provided a net benefit across a threshold probability range of 1% to 74% compared to the treat-all or treat-none strategies, with superior clinical utility in the low-to-intermediate probability range (Fig. 3G-I). As shown in Fig. 3H, if the threshold probability is set to 14% (i.e., the intervention will be performed on patients who have a predicted probability of AKI of > 14%), then 6 of 100 patients will receive a net benefit when using the nomogram.

### Patient characteristics before and after propensity-score matching

Of the total 673 patients, 23 were excluded due to loss to follow-up, leaving 650 patients included in the propensity score analysis. These patients were divided into an AKI group (*n* = 82) and a non-AKI group (*n* = 568). Before PSM, substantial between-group imbalances were observed, with SMD > 0.1 for multiple baseline covariates, including age, baseline NIHSS score, hypertension, diabetes, AF, Cancer, baseline renal insufficiency, onset to puncture time, Successful reperfusion, sICH, pneumonia, AHF, and hypotension. After PSM, 64 pairs of patients with similar baseline characteristics were successfully matched (Supplementary Fig. S3). In the matched cohort, most baseline covariates achieved adequate balance (SMD < 0.1), indicating a substantial reduction in between‑group differences (Table 3; Supplementary Fig. S4).

**Table 3:** Baseline characteristics of the patients before and after PSM

Variables	Before PSM			After PSM		
	Non-AKI (*n* = 568)	AKI (*n* = 82)	SMD	Non-AKI (*n* = 64)	AKI (*n* = 64)	SMD
Age, years	64.5 (55–74)	73 (59–82)	0.492	67 (58.3–76)	72.5 (59–82)	0.233†
Sex, n (%)			0.012			0.047
Female	187 (32.9%)	26 (31.7%)		22 (34.4%)	19 (29.7%)	
Male	381 (67.1%)	56 (68.3%)		42 (65.6%)	45 (70.3%)	
Baseline NIHSS score	14 (9–20)	20 (14–26)	0.613	20 (14, 22.8)	20 (14, 25.5)	0.046
Vascular occlusion site, n (%)			0.058			0.047
Anterior circulation	490 (86.3%)	66 (80.5%)		46 (71.9%)	49 (76.6%)	
Posterior circulation	78 (13.7%)	16 (19.5%)		18 (28.1%)	15 (23.4%)	
Thrombolysis, n (%)	123 (21.7%)	13 (15.9%)	0.058	12 (18.8%)	13 (20.3%)	0.016
Hypertension, n (%)	397 (69.9%)	67 (81.7%)	0.292	47 (73.4%)	51 (79.7%)	0.142†
Diabetes, n (%)	179 (31.5%)	36 (43.9%)	0.168	21 (32.8%)	25 (39.1%)	0.052
CHD, n (%)	79 (13.9%)	21 (25.6%)	0.005	16 (25.0%)	16 (25.0%)	0.016
AF, n (%)	164 (28.9%)	38 (46.3%)	0.118	25 (39.1%)	30 (46.9%)	0.063
Cancer, n (%)	17 (3.0%)	5 (6.1%)	0.124	2 (3.1%)	4 (6.3%)	0.063
Baseline renal Insufficiency, n (%)	98 (17.3%)	35 (42.7%)	0.117	23 (35.9%)	22 (34.4%)	0.000
Onset to puncture time, hours	6.08 (3.8–10.0)	5.4 (4.0-8.6)	0.175	4.8 (3.6–9.9)	5.5 (3.3–8.3)	0.078
Puncture to recanalization time, hours	0.5 (0.3–0.9)	0.6 (0.4–1.2)	0.031	0.6 (0.3–1.2)	0.5 (0.3–1.2)	0.031
Successful reperfusion, n (%)	550 (96.8%)	79 (96.3%)	0.254	62 (96.9%)	63 (98.4%)	0.016
SAH, n (%)	10 (1.8%)	7 (8.5%)	0.068	4 (6.3%)	4 (6.3%)	0.000
sICH, n (%)	51 (9%)	16 (19.5%)	0.105	10 (15.6%)	14 (21.9%)	0.063
Pneumonia, n (%)	171 (30.1%)	70 (85.4%)	0.553	51 (79.7%)	53 (82.8%)	0.031
AHF, n (%)	29 (5.1%)	36 (43.9%)	0.388	20 (31.3%)	20 (31.3%)	0.000
Hypotension, n (%)	41 (7.2%)	24 (29.3%)	0.220	18 (28.1%)	16 (25.0%)	0.031

Notes: † indicates residual imbalance after matching (SMD > 0.1)

Abbreviations: PSM, propensity score matching; SMD, standardised mean difference; AIS, acute ischemic stroke; AKI, acute kidney injury; NIHSS, National Institute of Health Stroke Scale; CHD, coronary heart disease; AF, atrial fibrillation; SAH, subarachnoid hemorrhage; sICH, symptomatic intracranial hemorrhage; AHF, acute heart failure

### Outcomes

**Fig. 4:**
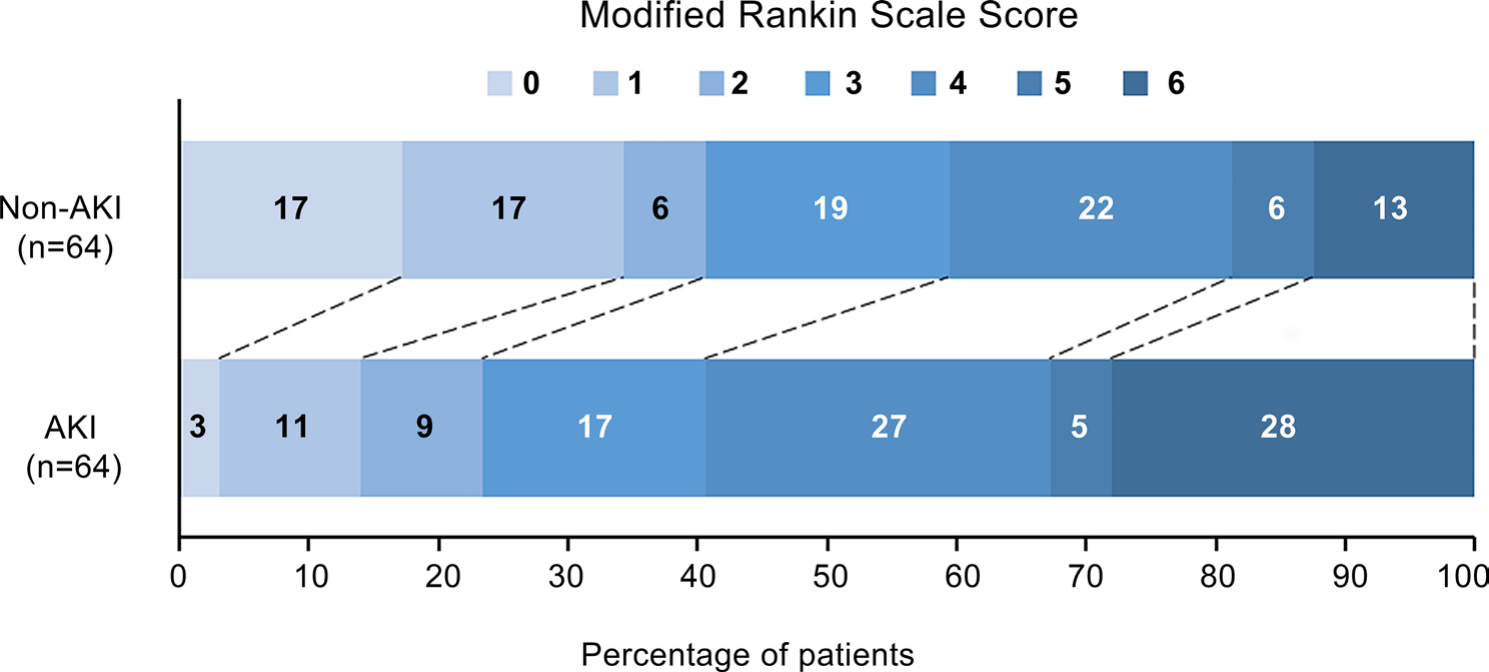
90-day mRS distribution in the matched data

As shown in Fig. 4 and Table 4, following PSM, the ordinal logistic regression model for the mRS outcome satisfied the proportional odds assumption (Supplementary Table S6), with an adjusted common odds ratio (cOR) of 2.322 (95% CI: 1.239–4.401, *P* = 0.01), indicating significantly worse neurological functional outcomes in AKI patients. The AKI group also showed higher rates of 90-day adverse functional outcomes (OR: 2.07, 95% CI: 0.94–4.68, *P* = 0.074) and 90-day mortality (OR: 2.42, 95% CI: 0.93–6.70, *P* = 0.076) compared to the non-AKI group, although these differences did not reach statistical significance. The pre-matching distribution of mRS scores and the univariate analysis results for the outcomes are presented in Supplementary Fig. S5 and Table S7, respectively.

**Table 4:** Analysis of 90-day mortality and adverse functional outcomes after PSM

Cohort	AKI Group, *n* (%)	Non-AKI Group, *n* (%)	Adjusted cOR/OR (95% CI)	*P*-value
Distribution of mRS Scores, n (%)			2.322 (1.239–4.401)	0.010
0	4 (6.3)	2 (3.1)		
1	7 (10.9)	3 (4.7)		
2	15 (23.4)	10 (15.6)		
3	12 (18.8)	14 (21.9)		
4	18 (28.1)	20 (31.3)		
5	8 (12.5)	15 (23.4)		
6	8 (12.5)	18 (28.1)		
Binary outcomes, n (%)				
90-day adverse functional outcome (mRS ≥ 3)	38 (59.4)	49 (76.6)	2.07 (0.94–4.68)	0.074
90-day mortality	8 (12.5)	18 (28.1)	2.42 (0.93–6.70)	0.076

Notes: The common odds ratio (cOR) for the ordinal shift analysis was derived from an ordinal logistic regression model. Binary outcomes were analyzed using multivariable logistic regression. All models were adjusted for age and onset-to-puncture time

Abbreviations: AKI, acute kidney injury; mRS, modified Rankin Scale; cOR, common odds ratio; OR, odds ratio; CI, confidence interval

## Discussion

The early symptoms of AKI in AIS patients undergoing EVT are often subtle, resulting in delayed treatment. This delay can lead to irreversible damage to kidney structure and function. Therefore, developing a risk prediction model to identify high-risk AKI patients is critically important.

In this study, five significant risk factors for AKI in AIS patients undergoing EVT were identified: baseline NIHSS score, preoperative creatinine level, pneumonia, AHF, and hypotension. Based on these factors, a nomogram was developed to predict the occurrence of AKI. The nomogram exhibited excellent discrimination, calibration, and high clinical utility, as evidenced by the AUC, calibration plots, and DCA in both internal and external validation cohorts. This nomogram can serve as a practical tool for clinicians to assess the risk of AKI in individual patients, facilitating personalized treatment strategies and early interventions.

Notably, in our model, the contrast agent dose was not selected as a predictor by LASSO regression. Moreover, in the external validation cohort—the most recent cohort with a significantly higher rate of preoperative CTA implementation—the incidence of AKI did not show a corresponding increase despite the additional accumulation of contrast agent dosage. This clinical observation is consistent with a growing body of evidence indicating a low incidence of contrast-induced nephropathy (CIN) following endovascular thrombectomy [21, 22]. Hence, contrast load appears to play a comparatively limited role in the multifactorial pathogenesis of post-EVT AKI, especially when weighed against other factors such as baseline stroke severity (NIHSS score) and hemodynamic instability.

Our findings revealed that a higher baseline NIHSS score was associated with an increased risk of AKI. Previous studies have demonstrated that brain injury can rapidly activate inflammatory cytokines, contributing to kidney inflammatory damage [4]. Additionally, brain injury may activate the SNS and RAAS, disrupting the central autonomic nervous system and the automatic regulation of renal blood flow. This disruption can lead to renal hypoperfusion and a reduction in GFR, potentially worsening kidney function [23]. For AIS patients with large-vessel occlusion, clinicians should closely monitor renal function and minimize the use of nephrotoxic medications whenever possible. Furthermore, a significant proportion of AIS patients (20% to 35%) had pre-existing renal impairment [24]. Our study identified a correlation between higher baseline creatinine levels and an increased risk of AKI following EVT, findings that align with those of previous studies [25, 26]. Based on these results, we recommend routine assessment of baseline renal function to evaluate the risk of AKI in patients undergoing EVT whenever feasible.

Both pneumonia and AHF are common complications in patients with AIS, and our study highlighted their association with AKI. Pneumonia triggers a pronounced systemic inflammatory response, which exerts a “second hit” on the already activated inflammatory state in patients with recent AIS, further aggravating intrarenal endothelial dysfunction. Additionally, pulmonary infection often leads to hypoxemia and/or hypercapnia, which directly activates the sympathetic nervous system (SNS), resulting in renal vasoconstriction and reduced renal blood flow. Cerebral hypoxia itself can exacerbate systemic sympathetic overactivation and hemodynamic instability, thereby forming a vicious cycle with those above local renal mechanisms and significantly increasing the risk of AKI [4, 27]. The occurrence of AHF can cause decreased cardiac output and venous congestion, resulting in decreased arterial perfusion of organs, and increased renal vein and renal interstitial pressure, thus inducing acute renal dysfunction [28, 29]. Moreover, hypotension often occurs in patients after EVT, which can activate the SNS and RAAS. This activation leads to the release of norepinephrine, epinephrine, and angiotensin II, resulting in renal artery constriction and thus reduced renal blood flow [8, 30]. Therefore, active measures should be taken to prevent pneumonia, AHF, and hypotension in AIS patients.

Previous studies have explored the risk factors of AKI. NIHSS score, preoperative creatinine level, hypotension, heart failure, and pneumonia have also been reported as associated factors [27, 31–33]. However, for AIS patients undergoing EVT, most previous studies primarily focused on identifying risk factors of AKI without establishing a clinically specific AKI prediction model, making it challenging to accurately quantify the risk of AKI [5, 25]. While a few studies have attempted to construct predictive models for AKI, such as those by Liu et al. [34] and Zhu et al. [35], these models have certain limitations. For instance, Liu et al. primarily focused on blood glucose levels and did not account for the impact of patient complications on AKI. Zhu et al. utilized data from the MIMIC database to assess AKI risk in AIS patients in the intensive care unit, but their analysis lacked information on surgical details and complications. Furthermore, the prediction models developed in these studies have not been validated in external cohorts. In contrast, our study comprehensively incorporated factors related to AKI before, during, and after interventional surgery. By employing LASSO regression and multivariate regression analysis, we identified risk factors with a strong correlation with AKI while excluding those with weak associations. After external validation, we developed a risk prediction model with high predictive performance and robustness.

Previous studies have consistently demonstrated the association between AKI and mortality [5, 12, 36]. However, The current evidence for an association between AKI and adverse functional outcomes in stroke patients is inconclusive. Yoo et al. [5] and Fandler-Hofler et al. [37] reported positive correlations between AKI and 90-day adverse functional outcomes using multivariate logistic regression, while Laible et al. [12] found no significant association despite employing the same methodology. These discrepancies may stem from differences in study populations and inadequate control of confounding factors. To address these limitations and more accurately assess the association between AKI and outcomes, our study utilized a PSM approach to minimize confounding bias. The results demonstrated that PSM significantly reduced the imbalance in baseline characteristics between the AKI and non-AKI groups compared with the prematched dataset. After matching, we found that AKI was significantly associated with worse neurological outcomes on the ordinal mRS scale (adjusted cOR 2.322, 95% CI: 1.239–4.401, *P* = 0.01), while also observing consistent—though not statistically significant—increases in the risks of 90-day adverse functional outcome (mRS ≥ 3) and mortality. This finding highlights the need to establish an integrated brain-kidney diagnosis and treatment pathway and provides important insights for future research in this field.

In summary, the poor outcomes associated with AKI in AIS patients undergoing EVT underscore the need for a tailored AKI risk prediction tool. Such a tool aids clinicians in accurately predicting the risk of AKI and guides clinical decision-making. Our study innovatively developed a user-friendly yet robust predictive tool to evaluate the probability of AKI following emergency EVT in AIS patients, which holds significant clinical significance. To enhance the practical application of this tool, we also established an online dynamic nomogram calculator (https://wdynamic.shinyapps.io/DynNomapp/). In this web interface, the probability of AKI can be accurately obtained by inputting the values of risk factors for AKI.

However, our study has several limitations. First and foremost, this was a single-center investigation. Consequently, the generalizability of our findings may be limited, as the patient population and management protocols might not be fully representative of other settings. Secondly, despite successful external validation, the significant differences in baseline characteristics and treatment protocols between the training and validation cohorts may affect the interpretation of the model’s performance. Thirdly, the application of PSM, while reducing confounding, also diminished the sample size in each group. This may have impacted the statistical power of our analysis and the overall representativeness of the results. Therefore, future large-scale, prospective, multi-center studies are warranted to validate the efficacy of this nomogram in broader clinical settings and to confirm our conclusions.

## Conclusion

We have developed an online dynamic nomogram that accurately predicted the risk of AKI in AIS patients undergoing EVT based on five key risk factors. The nomogram was user-friendly in clinical practice, facilitating the early identification of potential AKI patients and thereby assisting in the timely prevention and intervention of AKI occurrence. Furthermore, our study demonstrates that AKI after EVT in AIS patients is significantly associated with worse 90-day neurological functional outcomes.

## Supplementary Information

Below is the link to the electronic supplementary material.

Supplementary Material 1

## Data Availability

The data that support the findings of this study are available from the corresponding author.

## Abbreviations

ACEI: Angiotensin Converting Enzyme Inhibitors
AF: Atrial Fibrillation
AHF: Acute Heart Failure
AIS: Acute Ischemic Stroke
AKI: Acute Kidney Injury
ARB: Angiotensin II Receptor Blockers
AUC: Area Under The Curve
BUN: Blood Urea Nitrogen
CHD: Coronary Heart Disease
CI: Confidence Interval
CIN: Contrast-Induced Nephropathy
cOR: Common Odds Ratio
CTA: Computed Tomography Angiography
DCA: Decision-Curve Analysis
DBP: Diastolic Blood Pressure
eGFR: Estimated Glomerular Filtration Rate
EVT: Endovascular Therapy
HGB: Hemoglobin
HL: Hosmer-Lemeshow
IQR: Interquartile Range
LASSO: Least Absolute Shrinkage and Selection Operator
LVO: Large Vessel Occlusion
LY: Lymphocyte Count
MRA: Magnetic Resonance Angiography
mRS: Modified Rankin Scale
mTICI: Modified Thrombolysis in Cerebral Infarction
NEC: Neutrophil Count
NIHSS: National Institutes of Health Stroke Scale
OR: Odds Ratio
PLT: Platelet Count
PSM: Propensity-Score Matching
RAAS: Renin-Angiotensin-Aldosterone System
RBC: Red Blood Cell Count
RCS: Restricted Cubic Splines
ROC: Receiver Operating Characteristic Curve
SAH: Subarachnoid Hemorrhage
SBP: Systolic Blood Pressure
sICH: Symptomatic Intracranial Hemorrhage
SMD: Standardised Mean Difference
SNS: Sympathetic Nervous System
VIF: Variance Inflation Factors
WBC: White Blood Cell Count
