# Supplementary material for: An online dynamic nomogram for predicting acute kidney injury after endovascular therapy in acute ischemic stroke
Source: BMC Nephrol. 2026 Jan 27;27:127. doi: 10.1186/s12882-026-04773-9 (PMC12918098; doi:10.1186/s12882-026-04773-9)
Supplement: Supplementary file 1 — Supplementary Material 1 [file 12882_2026_4773_MOESM1_ESM.docx]

**Supplementary Materials**


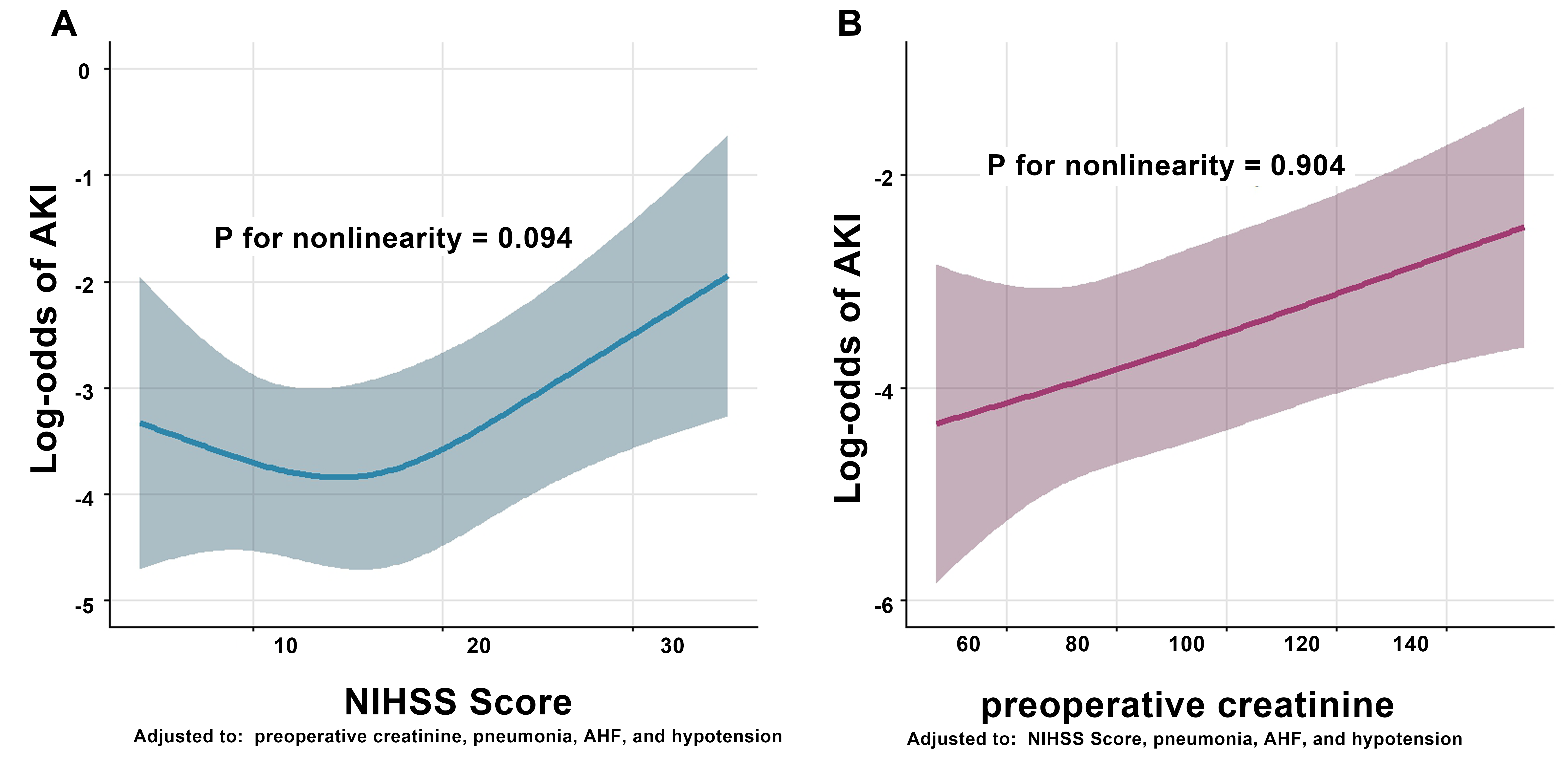


**Supplementary Fig. S1** Linear validation of the multivariate logistic regression incorporating baseline NIHSS score and creatinine.


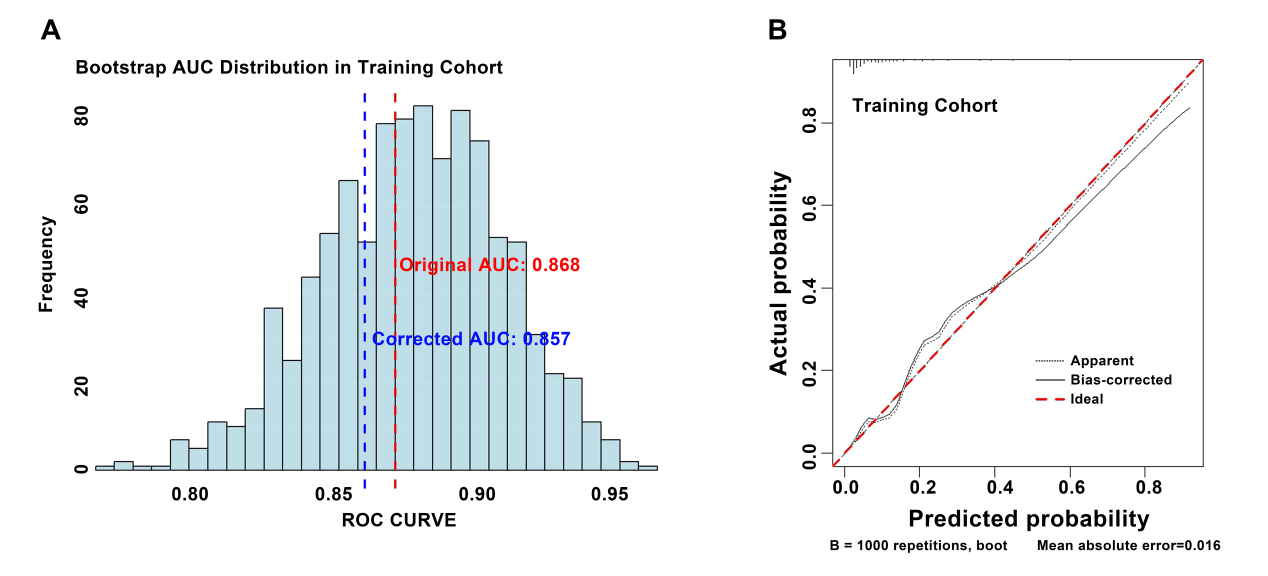


**Supplementary Fig. S2** Bootstrap calibration analysis of the training cohort. A: Receiver operating characteristic (ROC) distribution of the nomogram's predictive performance in the training cohort after internal bootstrap validation. B: Calibration curve of the nomogram in the training cohort after internal bootstrap validation. The red dashed line represents the reference line of an ideal model. The black solid line indicates the predicted probability by the nomogram; closer fit to the reference line denotes better calibration.


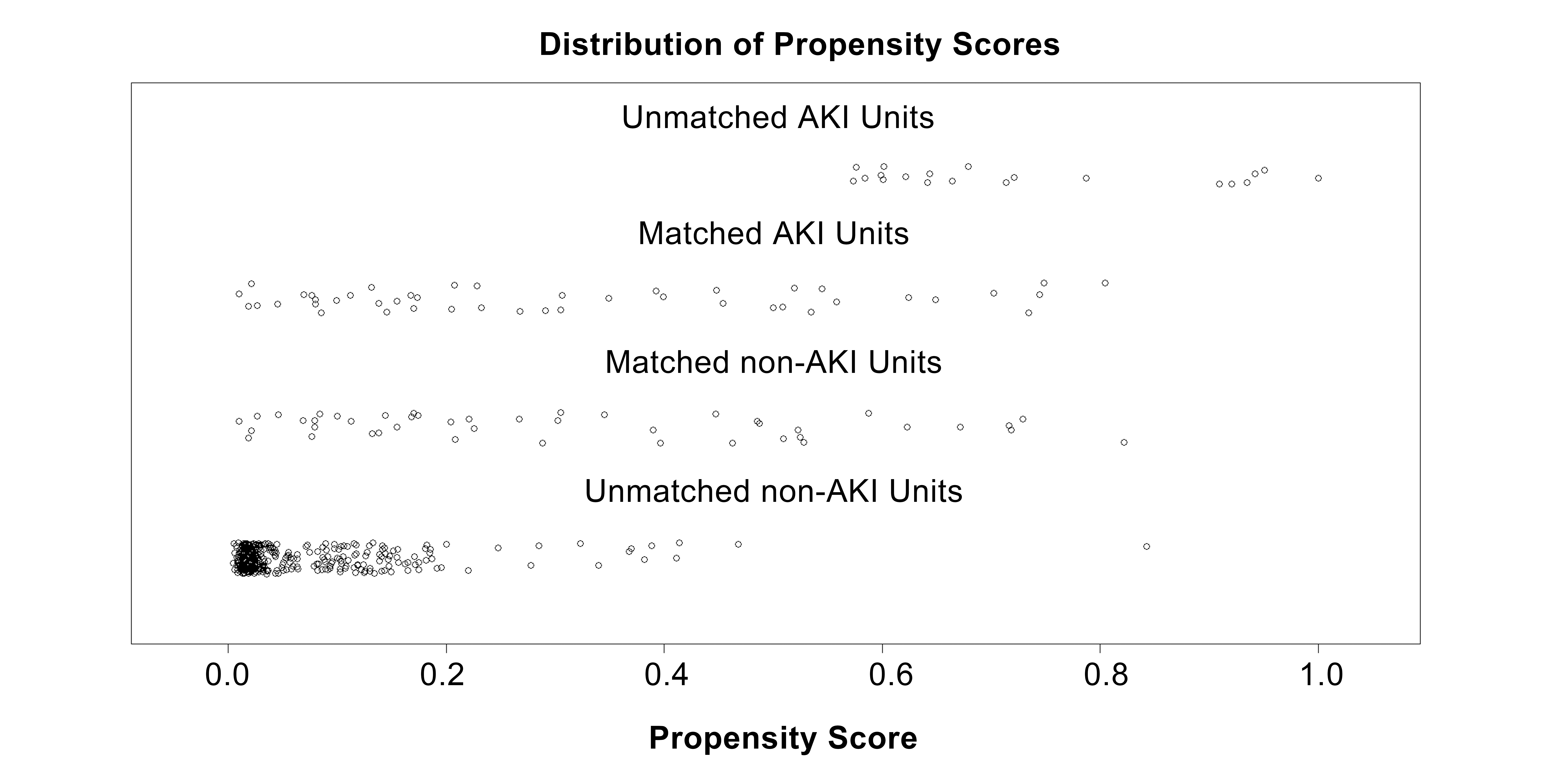


**Supplementary Fig. S3** Distribution of propensity score of matched and unmatched patients in the two groups.


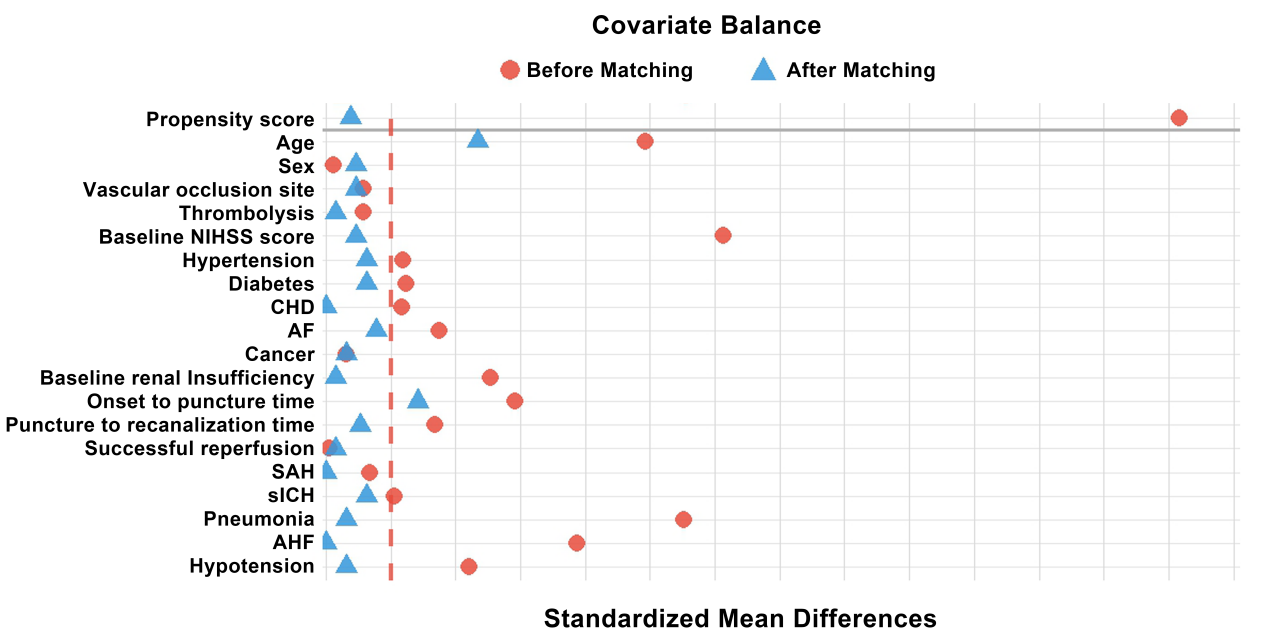


**Supplementary Fig. S4** Standardized Mean Differences of Covariates Before and After Propensity Score Matching.


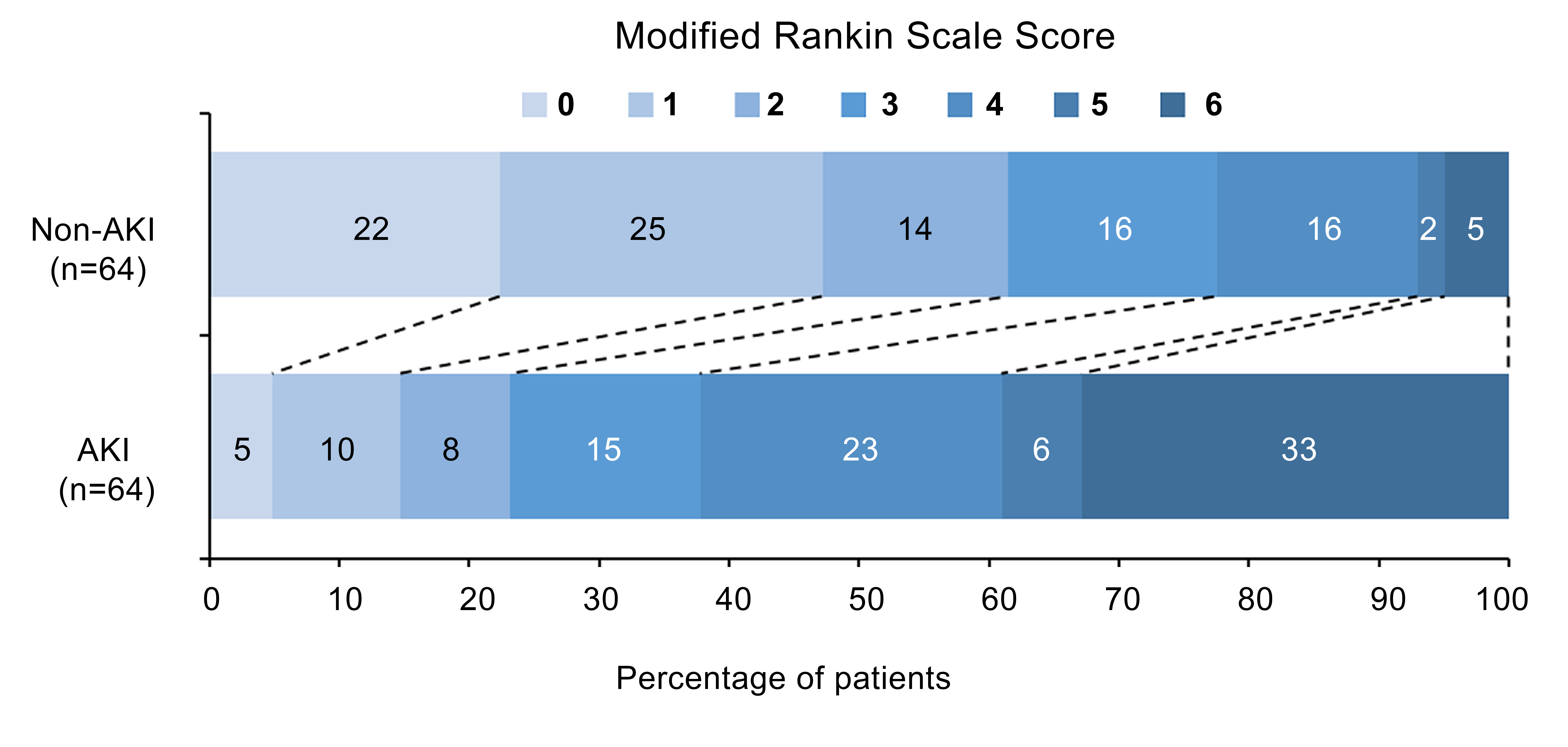


**Supplementary Fig. S5** 90-day mRS distribution in the prematched data.

| **Supplementary Table S1. CTA Utilization Across the Study Cohorts** | | | | |
| --- | --- | --- | --- | --- |
| Cohort | Group | CTA Performed, n (%) | CTA Not Performed, n (%) | *P*-value |
| Training cohort | Total | 107 (33.0) | 217 (67.0) |  |
|  | AKI | 11 (22.9) | 37 (77.1) | 0.134 |
|  | Non-AKI | 96 (34.8) | 180 (65.2) |  |
| Internal Validation | Total | 43 (30.7) | 97 (69.3) |  |
|  | AKI | 4 (23.5) | 13 (76.5) | 0.686 |
|  | Non-AKI | 39 (31.7) | 84 (68.3) |  |
| External Validation | Total | 158 (75.6) | 51 (24.4) |  |
|  | AKI | 13 (68.4) | 6 (31.6) | 0.445 |
|  | Non-AKI | 145 (76.3) | 45 (23.7) |  |

Note: The higher CTA rate in the external validation cohort resulted from the implementation of a standardized imaging protocol during 2022–2024, which routinely included multiphase CTA or CTP.

Abbreviations: CTA, computed tomography angiography; CTP, computed tomography perfusion.

| **Supplementary Table S2. Distribution of AKI Stages and Time of Onset Across Study Cohorts** | | | | | |
| --- | --- | --- | --- | --- | --- |
| Cohort | AKI Stage Distribution | | | Time of AKI Onset | |
|  | Stage 1, n(%) | Stage 2, n(%) | Stage 3, n(%) | 48h, n(%) | 72h, n (%) |
| Training cohort | 37 (77.1) | 9 (18.8) | 2 (4.2) | 22 (45.8) | 30 (62.5) |
| Internal validation | 14 (82.4) | 3 (17.6) | 0 (0) | 10 (58.8) | 12 (70.6) |
| External validation | 15 (78.9) | 3 (15.8) | 1 (5.3) | 9 (47.4) | 10 (52.6) |
| *p*-value |  |  | 0.922 | 0.6478 | 0.537 |

Notes: AKI stage was classified according to KDIGO (Kidney Disease: Improving Global Outcomes) guidelines.

Abbreviations: AKI, acute kidney injury; KDIGO, Kidney Disease: Improving Global Outcomes.

| **Supplementary Table S3. Missing Data for Baseline Characteristics in the Training, Internal Validation, and External Validation Cohorts** | | | |
| --- | --- | --- | --- |
| Variables | Training  cohort (n=324) | Internal validation cohort (n=140) | External validation cohort (n=209) |
| Age | 0 (0) | 0 (0) | 0 (0) |
| Sex | 0 (0) | 0 (0) | 0 (0) |
| Smoking status | 0 (0) | 0 (0) | 0 (0) |
| Baseline NIHSS score | 0 (0) | 0 (0) | 0 (0) |
| Preoperative SBP | 6 (1.85) | 2 (1.43) | 6 (2.87) |
| Preoperative DBP | 6 (1.85) | 2 (1.43) | 6 (2.87) |
| Vascular occlusion site | 0 (0) | 0 (0) | 0 (0) |
| Thrombolysis | 0 (0) | 0 (0) | 0 (0) |
| **Comorbidities** |  |  |  |
| Hypertension | 0 (0) | 0 (0) | 0 (0) |
| Diabetes | 0 (0) | 0 (0) | 0 (0) |
| CHD | 0 (0) | 0 (0) | 0 (0) |
| AF | 0 (0) | 0 (0) | 0 (0) |
| Cancer | 0 (0) | 0 (0) | 0 (0) |
| Baseline renal insufficiency | 0 (0) | 0 (0) | 0 (0) |
| **Preoperative laboratory data** |  |  |  |
| WBC | 2 (0.62) | 2 (1.43) | 2 (0.96) |
| NEC | 2 (0.62) | 2 (1.43) | 2 (0.96) |
| LY | 2 (0.62) | 2 (1.43) | 2 (0.96) |
| RBC | 2 (0.62) | 2 (1.43) | 2 (0.96) |
| HGB | 2 (0.62) | 2 (1.43) | 2 (0.96) |
| PLT | 2 (0.62) | 2 (1.43) | 2 (0.96) |
| Creatinine | 0 (0) | 0 (0) | 0 (0) |
| BUN | 1 (0.31) | 0 (0) | 0 (0) |
| eGFR | 0 (0) | 0 (0) | 0 (0) |
| Blood glucose | 1 (0.31) | 1 (0.71) | 0 (0) |
| **Surgical data** |  |  |  |
| Onset to puncture time | 6 (1.85) | 1 (0.71) | 0 (0) |
| Puncture to recanalization time | 8 (2.47) | 3 (2.14) | 0 (0) |
| Successful reperfusion | 0 (0) | 0 (0) | 0 (0) |
| Contrast dose | 26 (8.02) | 12 (8.57) | 5 (2.39) |
| Type of contrast agent | 0 (0) | 0 (0) | 0 (0) |
| **Treatment** |  |  |  |
| Antiplatelet | 0 (0) | 0 (0) | 0 (0) |
| Anticoagulants | 0 (0) | 0 (0) | 0 (0) |
| Statins | 0 (0) | 0 (0) | 0 (0) |
| Edaravone | 0 (0) | 0 (0) | 0 (0) |
| Antibiotics | 0 (0) | 0 (0) | 0 (0) |
| Mannitol | 0 (0) | 0 (0) | 0 (0) |
| Diuretics | 0 (0) | 0 (0) | 0 (0) |
| ACEI/ARB | 0 (0) | 0 (0) | 0 (0) |
| **Complications** |  |  |  |
| SAH | 0 (0) | 0 (0) | 0 (0) |
| sICH | 0 (0) | 0 (0) | 0 (0) |
| Pneumonia | 0 (0) | 0 (0) | 0 (0) |
| AHF | 0 (0) | 0 (0) | 0 (0) |
| Hypotension | 0 (0) | 0 (0) | 0 (0) |
| **Outcome** | 0 (0) |  |  |
| AKI | 0 (0) | 0 (0) | 0 (0) |

Notes: Data are presented as the number of missing cases (percentage).

Abbreviations: AIS, acute ischemic stroke; EVT, endovascular therapy; AKI, acute kidney injury; NIHSS, National Institute of Health Stroke Scale; SBP, systolic blood pressure; DBP, diastolic blood pressure; CHD, coronary heart disease; AF, atrial fibrillation; WBC, white blood cell count; NEC, neutrophil count; LY, lymphocyte count; RBC, red blood cell count; HGB, hemoglobin; PLT, platelet count; BUN, blood urea nitrogen; eGFR, estimated glomerular filtration rate; ACEI, angiotensin converting enzyme inhibitors; ARB, angiotensin II receptor blockers; SAH, subarachnoid hemorrhage; sICH, symptomatic intracranial hemorrhage; AHF, acute heart failure.

| **Supplementary Table S4. Multivariate logistic regression of the potential risk factors identified by LASSO regression** | | |
| --- | --- | --- |
| Variables | *P*-value | OR (95% CI) |
| Baseline NIHSS score | 0.030 | 1.063 (1.007-1.122) |
| Preoperative creatinine | <0.001 | 1.019 (1.009-1.031) |
| Diuretics use | 0.060 | 2.255 (0.954-5.318) |
| Pneumonia | 0.007 | 3.364 (1.412-8.526) |
| AHF | 0.003 | 3.842 (1.544-9.712) |
| Hypotension | 0.001 | 3.781 (1.359-10.432) |

Abbreviations: OR, odds ratio; CI, confidence interval; NIHSS, National Institute of Health Stroke Scale; AHF, acute heart failure.

| **Supplementary Table S5. Analysis of variance inflation factors for variables in the multivariate logistic regression model** | | |
| --- | --- | --- |
| Variables | VIF | Judgment |
| Baseline NIHSS score | 1.110 | No multicollinearity |
| Preoperative creatinine | 1.073 | No multicollinearity |
| Pneumonia | 1.107 | No multicollinearity |
| AHF | 1.026 | No multicollinearity |
| Hypotension | 1.031 | No multicollinearity |

Notes: A common threshold of VIF > 5 indicates severe multicollinearity. All VIF values in this study were well below 2, suggesting no multicollinearity among the independent variables.

Abbreviations: VIF, Variance Inflation Factor; NIHSS, National Institute of Health Stroke Scale; AHF, acute heart failure.

| **Supplementary Table S6. Brant test for the proportional odds assumption in ordinal logistic regression** | | | |
| --- | --- | --- | --- |
| Variables | χ² Value | Degrees of Freedom | *P*-value |
| Omnibus Test | 14.03 | 15 | 0.523 |
| AKI | 3.75 | 5 | 0.586 |
| Age | 2.15 | 5 | 0.828 |
| Onset to puncture time | 8.01 | 5 | 0.156 |

Notes: A non-significant *P*-value (*P* > 0.05) indicates that the proportional odds assumption holds. In this model, all *P*-values exceed 0.05, supporting the validity of the proportional odds model.

Abbreviations: AKI, acute kidney injury.

| **Supplementary Table S7. Analysis of 90-day mortality and adverse functional outcomes before PSM** | | | |
| --- | --- | --- | --- |
| Outcomes | Non-AKI | AKI | *P*-value |
| **Prematched data** |  |  |  |
| 90-day mortality | 28 (4.9%) | 27 (32.9%) | 0.000 |
| 90-day adverse functional outcome | 219 (38.5%) | 63 (76.8%) | 0.000 |

Abbreviations: PSM, propensity score matching.
